# Assessment of lower urinary tract symptoms during pregnancy: an observational cross-sectional study from Palestine

**DOI:** 10.1186/s12884-021-03567-1

**Published:** 2021-01-26

**Authors:** Jaffar H. Saffarini, Qais T. Ahmad, Ahmad M. Samara, Dima S. Jabri, Zaina H. Safarini, Yousra M. Banijaber, Ahmad Jaradat, Faris Abushamma, Sa’ed H. Zyoud

**Affiliations:** 1 Department of Medicine, College of Medicine and Health Sciences, An-Najah National University, Nablus, 44839 Palestine; 2 Department of Urology, An-Najah National University Hospital, Nablus, 44839 Palestine; 3 Bristol Urological Institute, North Bristol NHS Trust, Bristol, UK; 4 Department of Clinical and Community Pharmacy, College of Medicine and Health Sciences, An-Najah National University, Nablus, 44839 Palestine; 5 Poison Control and Drug Information Center (PCDIC), College of Medicine and Health Sciences, An-Najah National University, Nablus, 44839 Palestine; 6 Clinical Research Center, An-Najah National University Hospital, Nablus, 44839 Palestine

**Keywords:** Pregnancy, Lower urinary tract symptoms, Urinary incontinence, Palestine

## Abstract

**Background:**

Women frequently complain of lower urinary tract symptoms (LUTS) during pregnancy due to multiple physiological and potentially pathological changes. Still, there is limited data on the characteristics of LUTS for pregnant women in Palestine. Therefore, this study was designed to assess LUTS among pregnant women in Palestine, in addition to identifying factors that exacerbate LUTS during pregnancy.

**Methods:**

We devised a cross-sectional, questionnaire-based study that used the Urinary Distress Inventory (UDI) and the Incontinence Impact Questionnaire (IIQ) tools to assess LUTS during pregnancy in an antenatal care clinic setting. Multiple linear regressions were conducted to determine variables that significantly related to LUTS (UDI-6 and IIQ-7 scores as dependent variables).

**Results:**

The study recruited 306 pregnant women. Regarding LUTS findings, the participants scored a mean of 31.9 ± 24.9 out of 100 points (6.7 ± 5.2 out of 21) for the IIQ-7 scale and a mean of 31.2 ± 19.2 out of 100 points (5.6 ± 3.4 out of 18) for the UDI-6 scale. Regression analysis showed that older women (*p* = 0.031), women with a higher body mass index (*p* < 0.001), and women in the third trimester of pregnancy (*p* = 0.023), were independently associated with high UDI score. Multiple linear regression analysis showed that obese and overweight patients (*p* = 0.006) and multiparity (*p* = 0.026) were independently associated with high IIQ score.

**Conclusions:**

High body mass index is independently associated with both UDI and IIQ scores for LUTS. Several strategies should be arranged to raise the awareness of females of childbearing age in Palestine regarding LUTS during pregnancy and factors which may exacerbate LUTS, such as obesity and multiparity. Thus, preventive measures should be implemented, such as serial assessment of LUTS during antenatal care to respond timely to this frequent problem.

## Background

The urogenital apparatus undergoes multiple physiological and potentially pathological changes throughout pregnancy [1]. These changes may interfere with pregnant women’s daily activities and limit their social and physical lifestyle [2, 3]. Lower urinary tract symptoms (LUTS) is a broad term for a category of storage and voiding symptoms that affect both men and women. However, LUTS in women are slightly different from men, as overactive bladder syndrome and incontinence are more prevalent in females [4–10]. LUTS and related underlying conditions can cause major disruption to a pregnant female’s daily activities and interfere with their work, exercise, and social life [10–15]. Furthermore, LUTS can negatively impact the quality of life (QoL) of pregnant women [15, 16]. Thus, several strategies should be enacted during the antenatal period to identify LUTS. The level of awareness should be raised to encourage pregnant females to report their symptoms early, allowing proper evaluation and counselling [17].

LUTS during pregnancy can affect patients in a variety of ways. Possibly the most significant of these is the negative impact on QoL, as discussed previously [18, 19]. The impacts of LUTS on pregnant females encourages us, first of all, to try to identify factors that may exacerbate such symptoms, such as women’s age, body mass index (BMI), and multiparity. Secondly, clarifying indicators for LUTS helps with recognising risk factors prior to early pregnancy. This allows us to create education programs to raise awareness in childbearing females regarding risk factors and mitigate them through several strategies such as weight reduction programs and physical activity encouragement [11].

In Palestine, there is evidence that LUTS negatively affects pregnant women’s QoL [16]. Therefore, this study aimed to assess LUTS among pregnant women, in addition to identifying factors that exacerbate LUTS during pregnancy. The outcome of this study provides crucial clinical data on how LUTS during pregnancy affects women in Palestine.

## Methods

### Study design

Our study was an observational, cross-sectional, population-based study designed to assess the prevalence and severity of lower urinary tract symptoms, as well as factors associated with these symptoms, among pregnant women.

### Study setting and population

This study was conducted at Rafidia Hospital’s obstetrics and gynecology (OBGYN) clinic and targeted pregnant women between the ages of 18 and 40 years. The clinic visits for data collection were made between September 2019 and February 2020 during the official clinic hours (8 AM to 1 PM).

### Sample size and sampling technique

The overall number of pregnant women attending Rafidia Hospital’s OBGYN clinic was approximately 1100 during the study period. We used Raosoft online sample size calculator and obtained a sample size of around 300 based on our target population’s size. For sample selection, we adopted the convenience sampling method.

### Inclusion and exclusion criteria

The inclusion criteria comprised of pregnant women who can speak Arabic, were visiting Rafidia Hospital’s OBGYN clinic for pregnancy follow-up, were aged between 18 and 40 years, and had no previous diagnosis of a psychiatric disorder. We did not include all pregnant women because it is understood that a significant risk factor for labour and delivery complications is maternal age < 18 years or > 40 years [20, 21]. Moreover, as a general rule, in compliance with the Institutional Review Board (IRB), minors under 18 years cannot legally take their consent and require parent or guardian permission. In addition, our criteria excluded patients with a previous diagnosis of a urogenital condition, and patients having undergone urogenital surgery in the past.

### Data collection instrument

We performed data collection using an Arabic-language questionnaire. The questionnaire consisted of three parts. The first part inquired about demographic data, and the second and third parts contained the Incontinence Impact Questionnaire - Short Form (IIQ-7) and the Urinary Distress Inventory (UDI-6) [22, 23]; (we have requested permission to reproduce or reuse these scales by Wiley and Sons and Copyright Clearance Center: License Number 4987431113026).

In the first part, participants were asked to provide their demographic information, including age, type of residency, weight, height, smoking history, number of previous pregnancies, deliveries, and abortions, chronic psychiatric or medical conditions, chronic drug use, and surgical history. We classified participants into four age categories: 18–25 years, 26–30 years, 31–34 years, and 35 or more years. The BMI for pregnancy has been determined as pre-pregnancy weight in kilograms divided by height in square meters on the basis of self-reported weight and height. Participants were categorised based on their BMI into the underweight group (BMI < 18.5), healthy weight range group (BMI 18.5–24.9), overweight group (BMI 25–29.9), and obese group (BMI 30 or more).

The second part contained the UDI tool. The long version of UDI contains 19 items, covering 3 areas, and is designed to assess LUTS seriousness based on the level of bother caused over the previous month. In this study, however, we used a shorter version of this tool (UDI-6), which contains six items that cover the same three areas as the longer version: irritative symptoms (items 1 and 2), stress symptoms (items 3 and 4), and obstructive/discomfort symptoms (item 5 and 6). Each item had four response options: ‘not at all’, ‘a little bit’, ‘moderately’, and ‘greatly’. Each response to an item was given 0–3 points, with ‘not at all’ receiving 0 points and ‘greatly’ receiving 3 points, making the maximum total UDI score 18. The internal consistency of UDI was calculated using Cronbach’s alpha test and was found to have a coefficient of 0.720. Permission to use the instrument’s Arabic version was obtained from its developers [24].

The third part contained the IIQ tool, a widely accepted self-reported, contextual tool designed to assess life impact of incontinence in women with UI. The original version of this tool contains 30 items that cover four main topics. However, there is a shorter version of this questionnaire available (IIQ-7), consisting of seven items that cover the same four areas and is considered an acceptable alternative to the original 30-item version of the tool. The four topics that the IIQ-7 includes are: physical activity (items 1 and 2), travel (items 3 and 4), social relationships (item 5), and emotional health (items 6 and 7). In this study, we used the IIQ-7 version. The subject’s score was calculated after rating the severity of each symptom on a 0–3 scale for each symptom, with 0 for the least severe and 3 for the most severe [25]. The tool’s internal consistency was calculated before and was found to be high (Cronbach’s alpha coefficient of 0.894). Permission to use the tool’s Arabic version was also obtained from the developers. To allow comparison between scales, all scores for UDI-6 and IIQ-7 have been converted to a scale of 0–100 [26].

### Ethical approval

After we obtained approval from the *IRB of An-Najah National University* and the local health authorities, we proceeded with the study. The questionnaire included written informed consent for participants who read it and voluntarily accepted it before completing the question items.

### Statistical analysis

Data was entered and analysed by IBM SPSS, version 21 (IBM Inc., Armonk, NY). Participant characteristics were demonstrated as frequencies and percentages, and total scores on the tools used were expressed as medians and interquartile ranges. Variables were tested for normality using the Kolmogorov-Smirnov test. The Kruskal-Wallis test and the Mann-Whitney test were used to assess variations between various groups based on the participants’ characteristics. The significance was set at *p*-value < 0.05. Multiple linear regression was conducted to determine variables that were significantly related to LUTS assessment findings (UDI-6 and IIQ-7 scores as dependent variables). All variables with *p* < 0.05 in univariate analysis were used in the regression model. A variance inflation factor (VIF) has been checked for multicollinearity between the selected independent variables. Any values < 3 were deemed acceptable for lack of collinearity.

## Results

### Socio-demographic and clinical characteristics

In total, 323 pregnant women were approached to participate during the period of study. Out of the 323 pregnant women, 306 subjects who participated in our study completed the questionnaire (8 refused to participate and nine excluded based on inclusion and exclusion criteria), with a response rate of 94.7%. The age group that contained the largest percentages of participants (40.5%) was 18–25 years, and around 46% filled in the normal BMI group. Most of our subjects (71.2%) had had a university-level education. The majority also reported being housewives (77.8%), and living in a moderate-income household (48%). More than a third of the participants (41.2%) were in the third trimester of pregnancy, and most of them (70.6%) had had less than three children. Most of the women in our sample had no previously existing disease (91.8%) and did not perform a regular exercise (89.9%). The demographic and clinical characteristics of the participants are displayed in full in Table 1.

**Table 1:** Relationship between the participants’ characteristics and their UDI score and IIQ-7 score

Characteristic	Frequency (%); ***N*** = 306	UDI-6 score Median [Q1-Q3]	***P***-value*	IIQ-7 score Median [Q1-Q3]	***P***-value*
**Age category (years)**					
18–25	124 (40.5)	20.83 [8.33–32.29]	**0.033** ^**a**^	23.81 [9.52–52.38]	0.799 ^a^
26–30	113 (36.9)	25.00 [12.50–33.33]		33.33 [9.52–52.38]	
31–35	39 (12.7)	25.00 [20.83–41.67]		33.33 [9.52–47.62]	
> 35	30 (9.8)	25.00 [16.67–33.33]		33.33 [11.90–47.62]	
**BMI category**					
Healthy weight range	141 (46.1)	16.67 [8.33–27.08]	**< 0.001** ^**a**^	19.05 [0.00–47.62]	**0.002** ^**a**^
Overweight range	118 (38.6)	29.17 [16.67–37.50]		35.71 [14.29–52.38]	
Obese range	47 (15.4)	25.00 [16.67–37.50]		38.10 [19.05–57.14]	
**Education level**					
Elementary school	4 (1.3)	41.67 [16.67–57.29]	**0.042** ^**a**^	35.71 [29.76–63.10]	0.136 ^a^
Middle school	18 (5.9)	18.75 [8.33–33.33]		42.86 [25.00–48.81]	
High school	66 (21.6)	16.67 [8.33–33.33]		38.10 [17.56–52.38]	
University	218 (71.2)	25.00 [15.63–33.33]		26.19 [4.76–47.62]	
**Occupation**					
House wife	238 (77.8)	25.00 [12.50–33.33]	0.329 ^a^	33.33 [9.52–48.81]	0.302 ^a^
Government employee	31 (10.1)	20.83 [12.50–41.67]		38.10 [14.29–57.14]	
Private employee	37 (12.1)	16.67 [8.33–31.25]		23.81 [2.38–47.62]	
**Income**					
Low (< 2000 NIS)	123 (40.2)	25.00 [16.67–37.50]	0.092 ^a^	38.10 [14.29–52.38]	0.091 ^a^
Moderate (2000–4999 NIS)	147 (48)	20.83 [12.50–29.17]		28.57 [4.76–47.62]	
High (≥5000 NIS)	36 (11.8)	20.83 [9.38–29.17]		28.57 [4.76–47.62]	
**Gestational trimester**					
First trimester	68 (22.2)	16.67 [12.50–29.17]	**0.005** ^**a**^	19.05 [4.76–47.62]	0.146 ^a^
Second trimester	112 (36.6)	20.83 [12.50–33.33]		30.95 [9.52–47.62]	
Third trimester	126 (41.2)	25.00 [16.67–37.50]		38.10 [14.29–52.38]	
**Parity (number of deliveries)**					
< 3	216 (70.6)	20.83 [12.50–33.33]	0.285 ^b^	23.81 [4.76–47.62]	**0.002** ^**b**^
≥ 3	90 (29.4)	25.00 [12.50–37.50]		42.86 [27.38–52.38]	
**Previous diseases**					
No	281 (91.8)	20.83 [12.5–33.33]	0.281 ^b^	33.33 [7.14–47.62]	**0.035** ^**b**^
Yes	25 (8.2)	20.83 [14.58–31.25]		42.86 [21.43–57.14]	
**Drug history**					
No	287 (93.8)	25 [12.5–33.33]	0.639 ^b^	33.33 [9.52–47.62]	0.129 ^b^
Yes	19 (6.2)	20.83 [12.5–29.17]		38.10 [14.29–57.14]	
**Regular exercise**					
No	275 (89.9)	25.00 [12.50–33.33]	0.073 ^b^	33.33 [9.52–52.38]	0.279 ^b^
Yes	31 (10.1)	16.67 [9.33–25.00]		38.10 [9.52–52.38]	

*Abbreviations*: *UDI-6* Urinary Distress Inventory – Short Form, *IIQ-7* Incontinence Impact Questionnaire - Short Form, *BMI* body mass index, *NIS* New Israeli Shekel (1 NIS = 0.29 US Dollars)

* Significant *p*-values are in bold

^a^Calculated by using the Kruskal–Wallis test

^b^Calculated by using the Mann–Whitney U test

### Lower urinary tract symptoms assessment findings (UDI-6 and IIQ-7 scores)

Regarding LUTS findings, the participants scored a mean of 31.9 ± 24.9 out of 100 (6.7 ± 5.2 out of 21) points for the IIQ-7 scale and a mean of 31.2 ± 19.2 out of 100 (5.6 ± 3.4 out of 18) points for the UDI-6 scale. Table 2 summarizes the participants’ responses to the items in the UDI-6. More than a third of the participants (38.9%) were moderately affected by frequent urination, whereas 22.5% were greatly affected. A quarter of all participants reported that urine leakage was related to urgency (25.2%) or physical activity (24.9%) to a moderate or great extent. When asked if the urine leakage was small, 38.9% moderately agreed, and 22.5% agreed with that description. However, only 25.5% reported moderate-to-great difficulty urinating, and 24.9% experienced pain or discomfort.

**Table 2:** Distribution of responses to each question in the urinary distress inventory short-form (UDI-6)^a^ on a four-point Likert scale ranged from 0 to 3 (not at all, little, moderately, and greatly)

Do you experience and, if so, how much are you bothered by:	Not at All n (%)	A Little Bit n (%)	Moderately n (%)	Greatly n (%)
“Frequent Urination?”	62 (20.3)	56 (18.3)	119 (38.9)	69 (22.5)
“Urine leakage related to urgency?”	153 (50.0)	75 (24.5)	73 (23.9)	5 (1.6)
“Urine leakage related to physical activity?”	134 (43.8)	96 (31.4)	62 (20.3)	14 (4.6)
“Small amounts of urine leakage?”	62 (20.3)	56 (18.3)	119 (38.9)	69 (22.5)
“Difficulty emptying your bladder or difficulty urinating?”	153 (50.0)	75 (24.5)	73 (23.9)	5 (1.6)
“Pain or discomfort in your lower abdominal, pelvic, or genital area?”	134 (43.8)	96 (31.4)	62 (20.3)	14 (4.6)

^a^Adapted from Uebersax et al. 1995 [22]

Table 3 presents the participants’ responses to the questions in the IIQ-7 in detail. Only 7.2% of the participants felt that their ability to perform household chores was greatly affected, whereas 20.6% reported a moderate effect on their ability due to urine leakage. Physical recreation and entertaining activities were also greatly impacted by 5.2 and 4.6% of subjects and moderately impacted in 21.6 and 19.6% of the participants. A third of the participants (33.3%) felt a moderate-to-great effect of this symptom on their ability to travel for more than 30 min, whereas 28.8% reported the same impairment level (moderate-to-great effect) in their outdoors social functioning. When asked about the effect of urine leakage on their emotional health, 27.8 and 9.5% reported moderate and great effects, respectively.

**Table 3:** Distribution of responses to each question in the incontinence impact questionnaire, short-form (IIQ-7)^a^ on a four-point Likert scale ranged from 0 to 3 (not at all, slightly, moderately, and greatly)

Has urine leakage (incontinence) affected your:	Not at All n (%)	Slightly n (%)	Moderately n (%)	Greatly n (%)
“Ability to do household chores”	120 (39.2)	101 (33.0)	63 (20.6)	22 (7.2)
“Physical recreation such as walking, swimming, or other exercises?”	133 (43.5)	91 (29.7)	66 (21.6)	16 (5.2)
“Entertaining activities (movies, concerts, etc.)?”	154 (50.3)	78 (25.5)	60 (19.6)	14 (4.6)
“Ability to travel by car or bus more than 30 min from home?”	107 (35.0)	97 (31.7)	67 (21.9)	35 (11.4)
“Participation in social activities outside your home?”	115 (37.6)	103 (33.7)	74 (24.2)	14 (4.6)
“Emotional health (nervousness, depression, etc.)?”	102 (33.3)	90 (29.4)	85 (27.8)	29 (9.5)
“Feeling frustrated?”	140 (45.8)	99 (32.4)	40 (13.1)	27 (8.8)

^a^Adapted from Uebersax et al. 1995 [22]

### Pregnant women characteristics associated with UDI-6 and IIQ-7 scores

Table 1 shows the associations of the participants’ characteristics with their UDI score and IIQ-7 score, respectively. Many statistically significant associations were uncovered with both scores. UDI score was significantly associated with the participants’ age category (*p* = 0.033), BMI category (*p* < 0.001), education level (*p* = 0.042), and the trimester they were in (*p* = 0.005). We conducted a multiple linear regression analysis that was stratified according to age, BMI, educational level, and gestational trimester. UDI score was used as the dependent variable. Regression analysis showed that higher age groups (*p* = 0.031), obese or overweight pregnant (*p* < 0.001), and those pregnant women in their last gestational trimester (*p* = 0.023), were independently associated with high UDI score. The factors significantly associated with UDI based multiple linear regression analyses are listed in Table 4. There was no proof of multicollinearity between independent variables (VIF ranged from 1.018 to 1.059).

**Table 4:** Multiple linear regression analysis of the association between factors and Urogenital Distress Inventory (UDI-7)

Model		Unstandardized Coefficients		Standardized Coefficients	t	***P*** value*	95.0% Confidence Interval for B		Collinearity Statistics
		B	Std. Error	Beta			Lower Bound	Upper Bound	VIF
1	**(Constant)**	2.415	5.268		.458	0.647	−7.953	12.783	
	**Age**	1.780	0.821	0.119	2.167	**0.031**	0.164	3.396	1.021
	**BMI**	4.858	1.128	0.244	4.307	**< 0.001**	2.638	7.077	1.090
	**Education level**	1.784	1.199	0.082	1.488	0.138	−0.575	4.142	1.018
	**Gestational trimester**	2.367	1.037	0.128	2.284	**0.023**	0.327	4.407	1.059

*Bold values denote statistical significance at the *p* < 0.05 level

IIQ-7 score, on the other hand, was significantly associated with the participants’ BMI category (*p* = 0.002), parity (number of deliveries); (*p* = 0.002), and the presence of other chronic diseases (*p* = 0.035). The results of the multiple linear regression analysis, using IIQ score as a dependent variable and the covariates of BMI, parity (number of deliveries), and number of chronic diseases as independent variables showed that obese or overweight patients (*p* = 0.006), and parity (those with a greater number of deliveries) (*p* = 0.026), were independently associated with high IIQ score. Table 5 lists the variables significantly correlated with the IIQ score, based on multiple linear regression analyses. There was no proof of multicollinearity between independent variables (VIF ranged from 1.009 to 1.050).

**Table 5:** Multiple linear regression analysis of the association between factors and Incontinence Impact Questionnaire (IIQ-7)

Model		Unstandardized Coefficients		Standardized Coefficients	t	***P*** value*	95.0% Confidence Interval for B		Collinearity Statistics
		B	Std. Error	Beta			Lower Bound	Upper Bound	VIF
1	**(Constant)**	12.288	6.340		1.938	0.054	−0.188	24.765	
	**BMI**	5.347	1.940	0.155	2.756	**0.006**	1.530	9.164	1.009
	**Parity (number of deliveries)**	2.220	0.993	0.128	2.235	**0.026**	0.266	4.174	1.042
	**Previous diseases**	6.041	5.209	0.067	1.160	0.247	−4.209	16.291	1.050

*Bold values denote statistical significance at the *p* < 0.05 level

## Discussion

In addition to assessing LUTS among pregnant women in Palestine, the current study was planned to identify factors that worsen LUTS during pregnancy. Our study shows an association between the severity of urinary distress symptoms, which represents various LUTS (UDI-6 score), and the pregnant woman’s age. This finding is expected and predictable as several published articles showed that urinary incontinence and LUTS are more common in older women [27, 28].

Both UDI-6 and IIQ-7 scores were significantly associated with the BMI of pregnant women. Clearly, higher BMI exacerbates LUTS and especially incontinence in pregnant women. This has been a trending concept in the literature recently, as shown by several published articles. For instance, Yi-Hao Lin et al. showed a strong correlation between stress urinary incontinence and body weight and body mass index [29]. Furthermore, Zhiyi Li showed a mixture of voiding and storage LUTS during pregnancy directly associated with BMI. For example, a patient with a low BMI was more likely to have voiding LUTS during late pregnancy [14].

Our study shows that the stage of pregnancy is directly associated with the presence and severity of LUTS. In particular, the severity of LUTS as measured by the UDI-6 scale was significantly higher in women at the advanced stage of pregnancy than those still in the early pregnancy stage. This is supported by the available evidence that the late stage of pregnancy is associated with more severe LUTS symptoms [12].

Multiparity is another major factor that contributes to LUTS and incontinence, as supported by our results. This may be explained by the higher incontinence rate related to weak pelvic floor muscle in multipara women. Thus, pelvic floor muscle exercises should be highlighted after pregnancy, and education should be given to all childbearing women regarding this concept as a means to avoid this problem [30].

Finally, patient age, multiparity, obesity, and stage of pregnancy are all factors that, according to our study, influence LUTS during pregnancy. Therefore, education should be available to all women of childbearing age, to enable them to modify lifestyle risk factors or at least quickly recognise symptoms early during pregnancy to avoid the harmful effects, particularly on QoL [16, 30].

Among the limitations of this study was its cross-sectional design, which did not allow for making a causality interpretation of the associations we found. Moreover, the study’s unicentric nature might have limited our ability to generalise the data to all pregnant Palestinian women. However, our study population matches the average pregnant Palestinian woman (e.g., young age, normal BMI to overweight, university-level degree, housewives, parity < 3 children) [16, 31–34], meaning the study population adequately represents the Palestinian population, therefore it can be applied to most women in the region. On the other hand, the current study had many strength points, including using two urogenital assessment tools widely used and have well-established validity, which has helped achieve a more accurate assessment. Additionally, this was the first study to address factors that worsen LUTS during pregnancy in Palestine.

## Conclusions

LUTS are a prevalent problem during pregnancy, directly related to women’s age, gestational age, and parity. BMI is a major contributor to LUTS. Thus, preventive measures should be implemented, such as serial assessment of LUTS during antenatal care to respond timely to this frequent problem. Several strategies are needed to raise awareness for LUTS, including educational programs for females of childbearing age regarding LUTS during pregnancy. Further studies should involve larger samples and study the effect of LUTS on pregnancy and the overall health status of women.

## Data Availability

The datasets used and/or analyzed during the current study are available from the corresponding author on reasonable request.

## Abbreviations

BMI: Body mass index
IIQ: Incontinence Impact Questionnaire
IRB: Institutional Review Board
LUTS: Lower urinary tract symptoms
NIS: New Israeli Shekel
NNU: An-Najah National University
OBGYN: Obstetrics and gynecology
QoL: Quality of life
UI: Urinary incontinence
UDI: Urinary Distress Inventory
VIF: Variance inflation factor
